# First-generation immigrants’ experiences of entering and remaining in the Swedish labour market in a sustainable way: A qualitative study

**DOI:** 10.1177/10519815261431794

**Published:** 2026-03-18

**Authors:** Maria Brendler-Lindqvist, Magnus Svartengren, Martin Tondel, Therese Hellman

**Affiliations:** 1 Department of Medical Sciences, Occupational and Environmental Medicine, Uppsala University, Uppsala, Sweden; 2 Department of Occupational and Environmental Medicine, Uppsala University Hospital, Uppsala, Sweden

**Keywords:** unemployment, emigration and immigration, occupations, workplace, workforce diversity, quality of life, self efficacy

## Abstract

**Background:**

Integration of immigrants on the labour market is a pressing issue in most Western countries. However, while previous research has focused on immigrants getting their first job, less attention has been paid to the sustainability of immigrants’ labour market participation over time.

**Objective:**

The aim of the study was to explore first-generation immigrants’ experiences of entering and maintaining employment in the Swedish labour market in a sustainable way.

**Methods:**

Data was collected through individual semi-structured interviews with 18 non-European immigrants who lived in Sweden between 4 to38 years and who had experienced periods of work and unemployment. The education level of participants varied from four years of primary school to university degree. Interviews were analysed with thematic analysis applying an inductive approach.

**Results:**

Participants’ employment situations were characterised by persistent employment instability which had both practical and emotional consequences. In the process of establishing themselves in the labour market, participants had to navigate between personal aspirations, needs and contextual constraints, as well as struggle with feelings of frustration. Social support, both in the workplace and outside, was crucial in coping with the situation. The desire for a meaningful belonging to a workplace emerged as an important theme in the interviews.

**Conclusion:**

Persistent employment instability challenged immigrants’ professional identity, well-being and agency. To deal with this, support measures need to be flexible and provided both during periods of unemployment and while employed. Moreover, the role of the workplace in supporting immigrants’ sustainable labour market participation need to be strengthened.

## Introduction

Immigrants constitute an important share of the workforce in Western countries. Nevertheless, they are often found in disadvantaged positions in the labour market, with low salaries, unstable working arrangements and high unemployment rates.^1,2^ This is also the case in Sweden, where in 2025 approximately 20% of the population was born in another country.^3^ Despite immigrants’ labour market participation being a top priority of Sweden's integration policy for several decades, unemployment rates among immigrants remain high. In 2021, about 18.4% of the foreign-born population was unemployed, compared to 4.4% of Swedish-born individuals – a gap that is wider than in most Western countries.^4^

Previous research on immigrants’ labour market participation has mainly examined individual and country-level factors influencing employment. Studies show that refugee status, non-European origin, older age at immigration and lower education increases the risk of unemployment, while host-country language proficiency, education obtained in the new country and motivation improve job chances.^5–7^ Macroeconomic conditions at the time of immigration, local labour markets and ethnic discrimination are important structural factors affecting immigrants’ employment opportunities.^8–11^ Fewer studies have explored the process through which labour market integration takes place in practice, including the role of the work environment, employers’ attitudes, and the interaction of different stakeholders.^7,12–15^ Moreover, policy and research tend to emphasize entry into the first job, despite evidence that immigrants’ face a higher risk of re-enter unemployment even after labour market entry compared to non-immigrants with comparable qualifications.^16,17^

A sustainable working life, i.e., the ability to work until retirement in good health and to adapt to structural changes in the labour market over the life-course, is important for both individuals and society.^18^ For the individual, working life sustainability is important as employment provides an income, as well as a social context, a shared purpose with others and a professional identity.^19^ For society, utilizing immigrants’ competencies in the labour market are important for economic development, as well as social cohesion. However, the increasing flexibilization of labour markets and technological developments such as digitalization and artificial intelligence bring new challenges to working life and may threaten workers’ health and well-being.^20–22^ These rapid changes call for moving beyond the traditional focus on job-specific workability towards a broader understanding that also encompass long-term employability. The conceptual model of sustainable careers by de Vos et al. presents a useful framework in this regard. It highlights the interaction between the individual, the context, and dynamic changes over time and may also inform the understanding of immigrants’ labour market participation.^23^

In summary, while sustainable labour market participation among immigrants is a pressing issue, little is known about how the process of entering and remaining in the labour market unfolds in practice and how long-term sustainability can be supported. Moreover, there is a lack of research on how immigrants themselves experience this process. Thus, this study aimed to explore first-generation immigrants’ experiences of entering and remaining in the Swedish labour market in a sustainable way. The specific research questions where:

- How do participants experience the process of entering and remaining on the labour market?
- How do personal and contextual aspects interact to influence this experience?

## Method

### Recruitment and description of participants

The study is an exploratory qualitative study. Eligible participants were immigrants who came to Sweden as adults from non-Western countries and had experienced periods of employment and unemployment after immigration. Moreover, participants had to speak Swedish or English and express a desire to work. Purposeful sampling was used, meaning that the recruitment strategy actively aimed for a varied and information-rich sample with regard to the experiences we sought to examine.^24^ Information about the study was disseminated through immigrant organisations and support programmes for the long-term unemployed. Interested participants contacted the researcher, who provided further information about the study purpose, aim and method and collected signed informed consent documents. In total, 11 women and seven men were interviewed. Their ages ranged from 22 to 69 years at the time of the interview. All but two participants were of working age and were currently unemployed and seeking employment. Two participants had retired within the past three years; however, they were included as they had previous experiences of employment and unemployment in Sweden and could contribute to answering the research question. Among those participants who were included in the study no participant withdrew its participation. Participants came from the Middle East, Afghanistan and the Horn of Africa and had lived in Sweden for between 4 and 38 years. Most were under the age of 35 at the time of immigration, i.e., they were at the beginning, or in the midst, of their working careers. Two-thirds of the participants had children who lived in Sweden. Eight were married, while the remaining eight were either divorced or widowed (Table 1).

**Table 1.** Socio-demographic characteristics.

Participant characteristics, N = 18	N mean (range)
Women	11
Age	47 (22–69)
**Age at immigration**	
< 25 years	6
25–34 years	7
> 34 years	5
**Time in Sweden**	
0–10 years	5
>10 years	13
**Region of origin**	
Horn of Africa	6
Middle East and Afghanistan	12
**Family situation**	
Married	8
Divorced/widow	8
Having children who live in Sweden	12
**Highest attained education from country of origin^1^**	
Post-secondary education	8
Upper secondary education	4
Primary and lower secondary education or less	6

1) In case of uncertainty of highest attained level, the lower level was chosen.

**Source:** Authors own work

The participants’ education levels varied from four years of primary school to a university degree, and their educational backgrounds were within the areas of education science and teacher training, healthcare, social work, journalism, engineering, manufacturing and agriculture (Table 2). Some participants had extensive work experience before migrating, while others had not worked in their countries of origin, primarily because they arrived in Sweden at a young age (not shown in the Table). After migrating to Sweden, all participants had been in contact with the Swedish Public Employment Service (PES). The majority had studied the Swedish language at school after arrival, and several had undertaken vocational training in Sweden in the areas of healthcare and nursing, social work and welfare, hotel, restaurant and catering, engineering, construction and forestry. One participant had her teaching degree validated in order to obtain a Swedish teaching certificate. Work experience in Sweden included roles as teachers and native language teachers, interpreters, social workers, sports instructors, service and sales staff, childcare workers, teachers’ aides, personal assistants, home care workers, cleaners, construction labourers, restaurant and kitchen assistants. Employment was often in the form of short-term contracts and/or part-time jobs.

**Table 2.** Education and employment experiences of participants.

Participant	Highest educational level in country of origin ^1^	Main education undertaken in Sweden ^2^	Main work experiences in sweden^2^
**Women**			
1	Post-secondary education, engineering and manufacturing	Vocational training, mechanics	Teacher, social work, sales
2	Upper secondary education	Municipal adult education	Childcare worker, administration, cleaner
3	Post-secondary education, teacher training	Vocational training, healthcare and nursing	Services and sales, hairdresser, social work
4	Post-secondary education, teacher training	Fast track programme for teachers	Youth club, teachers’ aide
5	Post-secondary education, journalist	Vocational training, hotel and restaurant	Restaurant and kitchen assistant, personal assistant, teachers’ aide, social work, interpreter
6	Post-secondary education, social worker	Freestanding university course	Teacher, social worker, interpreter, administration
7	Primary education	Vocational training, healthcare and nursing	Personal assistant, study circle leader, assistant
8	Post-secondary education, agronomy	Vocational training, hotel and restaurant	Childcare worker, interpreter
9	Less than primary education	Vocational training, child and recreation	Cleaner, childcare worker
10	Post-secondary education, teacher training	Swedish teaching certification by validation	Service and sales, teacher, teachers’ assistant
11	Primary education	Vocational training, child and recreation	Childcare worker
**Men**			
12	Lower secondary education	Vocational training, forestry	Assistant teacher, warehouse worker, cleaner
13	Post-secondary education, dentistry		Construction labourer, restaurant and kitchen assistant, homecare worker
14	Upper secondary education		Gardener, warehouse worker
15	Less than primary education		Warehouse worker
16	Upper secondary education		Cleaner, restaurant and kitchen assistant
17	Lower secondary education	Vocational training, drilling technician	Restaurant and kitchen assistant, building caretaker, cleaner
18	Upper secondary education		Artist, sports instructor

1) In case of uncertainty, the lower level was chosen. 2) The list is not exhaustive.

**Source:** Authors own work

### Data collection

Data collection took place during autumn 2022. Individual interviews were held on one single occasion and lasted approximately 60 min (range 26–87 min). Interviews took place at the premises of an organisation, a support programme or the Department of Occupational and Environmental Medicine at Uppsala University Hospital, depending on participants’ preference. All interviews were held in a separate room with no one else present except from the participant and the interviewer. The interviews were conducted by M.B-L. who has previous knowledge in interview technique from clinical work as a medical doctor and with supervision from T.H. who has extensive experience in qualitative interview techniques. The interviews followed a semi-structured interview guide, covering experiences of working in Sweden and of being outside the labour market, as well as participants’ reflections on factors hindering or facilitating sustainable labour market participation. It was inspired by the conceptual model of sustainable careers by the Vos et al., focusing on the interplay between individual and contextual factors on the workplace and outside, as well as the labour market integration process over time^23^ (Supplementary_material_appendix_1). The first interview served also served as a pilot interview which resulted in smaller adjustments of the interview guide after discussions between M.B-L. and T.H. All interviews were conducted in Swedish, with participants exhibiting varying levels of proficiency in the language. Thus, to overcome any language barriers, the interviewer sometimes needed to simplify and rephrase questions or probe further to ensure mutual understanding. Data collection continued until no new information emerged in relation to the aim of the study which was discussed continuously between M.B-L. and T.H. All interviews were audio-recorded and transcribed verbatim. No field notes or other information was gathered during the interview occasions. Participants were informed that they may contact the interviewer after the interview if they wanted to add any information, however, this was not the case of any of the participants. In this study, participants were not offered the opportunity to review the transcripts or provide feed-back on the findings. This decision was made based on the assessment that a meaningful and thorough process of participant involvement would have been difficult to achieve given the variation in education level and Swedish language proficiency of the participants.

### Data analysis

Data were analysed inductively using thematic analysis, following the procedure proposed by Braun and Clarke.^25^ First, the transcribed interviews were read through to gain a sense of the overall meaning of the data. Second, data extracts were identified and coded, and the codes were subsequently grouped into potential themes for each interview. This step was conducted using Nvivo 14 software. The first interview was coded separately by two of the authors (M.B-L. and T.H.), and their coding was subsequently compared and discussed to ensure a common interpretation of the material and to confirm that the codes generated were not influenced by the researchers’ preconceptions. The subsequent interviews were coded by M.B-L., however, interpretations were discussed between M.B-L. and T.H. in case of ambiguities in how to interpret data. Following the separate coding of each interview, the individual-level codes were combined into aggregated codes which were subsequently organized into sub-themes and themes (Table 3). During the process, the first author (M.B-L.) went back and forth continuously between the transcribed full interviews, data extracts, codes and themes to ensure that the meaning was understood correctly. Feedback was provided by the research team during the process until a consensus was reached on the final themes. The research team consisted of two men and two women. Three of the authors were physicians within the area of occupational medicine (M.B-L., M.T. and M.S.), and one was an occupational therapist (T.H.). All authors had prior research experience in the area of working life.

**Table 3.** Data analysis process and findings.

Main theme	Employment instability over time challenge professional identity, well-being and sense of agency							
Themes	Coping with the practical and emotional consequences of unstable employment situations			Being part of a workplace		Balancing aspirations and needs amidst contextual constraints		
**Sub-themes**	Struggling with feelings of disappointment and frustration	Struggling with a lack of structure, economic hardships and concerns	Need for social support to maintain well-being, a positive self-image and professional identity	Feeling needed and capable	Being part of a social context	Adopting strategies to achieve goals within contextual constraints	Navigating aspirations and needs in relation to language skills	Making decisions based on limited information
Aggregated codes*	Uncertainty about reasons for unemployment Feeling exploited and unfairly treated Feelings of disappointment and frustration Reflections on possible discrimination	Financial and practical consequences Isolation Impact on health and well-being	Maintaining one's identity Coping strategies (activities, support, keeping hope) Experiences with PES	Feeling needed Experiencing work as meaningful Feeling capable for the work	Working environment: colleagues Working environment: supervisors	Stable job more important than job field Some jobs not considered Private-life barriers Work vs education priorities	Language is key for work and education Learning the language - a challenge Native language as job asset	Job-seeking flexibility as a strategy Personal network in job search Requested support in job search

*Codes resulting from aggregation of individual-level codes.

Source: Authors own work

### Ethical considerations

Participation was voluntary, and the study was approved by the Swedish Ethical Review Authority (File number2022-01757-01).

## Results

### Employment instability over time challenges professional identity, well-being and sense of agency

The employment situation of the participants was characterised by instability, with periods of unemployment alternating with short-term contracts, subsidised employment, or work as day-to-day substitutes. Many also held part-time jobs while actively searching for full-time employment. Moreover, this precarious situation persisted over time, despite more work experience, improved language skills and further education. The main theme identified was that this prolonged employment instability and lack of career progress constituted an ongoing challenge to professional identity, well-being and sense of agency, i.e., an individual's sense of control and ability to act.

Several participants described migration as “a start from zero”. They recognised that coming to a new country as an adult implied reduced opportunities compared to those who had studied in Sweden and learned the language from a young age. Some expressed that acceptance of this reality was a necessary first step in adapting and reorienting themselves. Most participants were refugees or family members of refugees. They described having only vague ideas about their future working lives before migrating, as their decision to migrate was driven by other factors. While they had envisioned working, they had little understanding of the professional opportunities available. As one participant noted:

*I plan to work in Sweden as usual, like other people do. A job that suits me, and that I can earn a little salary from.* (Participant 11, female)

However, even though most participants lacked concrete expectations regarding their future working life, they had not foreseen being stuck in unstable work arrangements, as was often the case. It emerged that, for the participants, the lack of improvement in their employment situation had a particularly negative impact. While several participants expressed being satisfied and happy with temporary or low-skilled jobs during their first years in Sweden, these feelings gradually changed. In addition, they raised concerns that increasing age and declining health might further limit job opportunities. The frustration caused by the lack of improvement or deterioration of their employment situation, despite efforts undertaken, is illustrated by the following quote:

*Yes, there are jobs in restaurants, or in moving and cleaning, [but I will] never do that. Why should I? I took courses; I struggled for two years. […] Why should I go back twenty years, [doing the kind of work I did when I first arrived in Sweden?]* (Participant 17, male)

**Figure 1.**
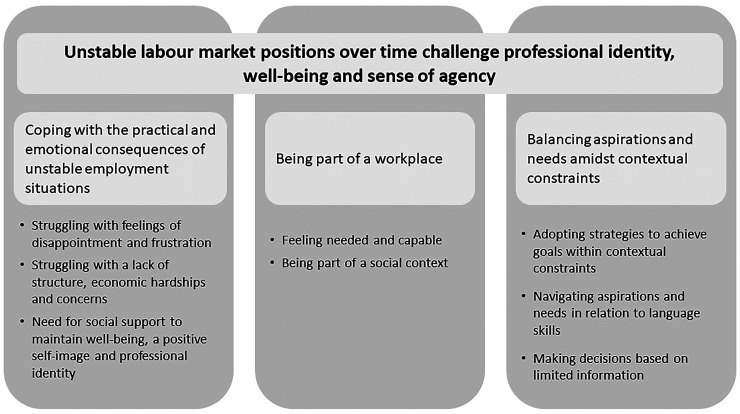
Illustration of the themes and sub-themes.

Thus, while participants expressed a willingness to adapt and reorient themselves in response to the difficulties of migration, the findings showed that when their situation failed to improve, or worsened over time, it became difficult to accept. Three themes were identified that elaborate on how the persistent employment instability and lack of career progression shaped different aspects of the participants’ working life experiences Figure 1.

### Coping with the practical and emotional consequences of unstable employment situations

Participants’ struggle to manage the practical and emotional consequences of employment instability is elaborated in the first two sub-themes. The third sub-theme describes the crucial role of social support in coping with these consequences, especially the emotional ones.

#### Struggling with feelings of disappointment and frustration

A common theme in many interviews was the feeling of disappointment and frustration when job applications were rejected or contracts were not renewed. This was especially evident when subsidised employment did not result in permanent employment. Participants often had high expectations for these positions, believing they had performed well and were needed and appreciated in the workplace, as expressed by this woman:

*You know, you feel hopeless because we want to work– I want to work. When you work, they say that you do a great job. I feel so happy, thinking, maybe this time I will get employed? But in the end, they just say “goodbye” to us.* (Participant 5, female)

Working on temporary contracts or as substitutes for extended periods was also experienced as frustrating. Participants often described feeling exploited by employers as, from their experiences, these positions did not enhance their chances of securing regular, permanent employment. Participants’ efforts to meet the demands of employers and enhance their skills without seeing any positive effect on their job prospects left them with a sense of uncertainty and hopelessness, as reflected below:

*Before, it was like, “you must have some kind of education to get a work”. So, I got an education, but… I thought that once I had an education, I would get a job, but I don’t know why [I don’t get a job].* (Participant 11, female)

Feelings of rejection may, in some cases, even lead to decreased motivation to continue searching for a job:

*Sometimes I get the feeling that society does not want anything for us. They play with us. Many people get depressed; they do negative things. If you lose your motivation, you just let things go. Really. Slowly, you feel that “ok, society does not want me to have a job, so I will not make an effort”.* (Participant 18, male)

Some participants mentioned ethnic discrimination by employers as a reason for not getting a job. However, most were uncertain about the presence of discrimination or reluctant to comment on the issue. For most participants, the reason for not getting a job was unclear, leaving them unsure of how to move forward and improve their situation.

#### Struggling with a lack of structure, economic hardships and concerns

Participants also highlighted consequences of unemployment, such as the loss of structure in daily life, isolation and financial hardships, and how these factors led to psychological stress and decline in well-being. To cope with these negative consequences of unemployment, participants stressed the importance of maintaining daily routines and staying active, for example, through physical activities, music, or visits to the library. Several also emphasized the need to remain optimistic about their chances of finding work and to not lose hope, as expressed by one participant:

“*I have to think that the job will come when it comes. Otherwise, it's a problem if I start thinking about other things, like why the job isn't coming (…). So, I try to think like that — when it comes, it comes.* (Participant 12, male)

However, some participants stressed that, over time, concerns about the future became an increasing threat to their well-being, as they feared that diminishing capabilities and declining health further reduced their chances of finding a job.

#### Need for social support to maintain well-being, a positive self-image and professional identity

The importance of practical and psychological support in coping with the negative consequences of unemployment was raised in the interviews and several participants stressed that family and friends were important in this regard.

The importance of a supportive relationship was also stressed in connection with support from the Swedish Public Employment Service (PES). All participants had experienced support initiatives through the PES or its providers, but their assessment of this support varied greatly. Those who were satisfied stressed the importance of regular contact with a personal employment officer/supervisor, whom they perceived as being engaged in their case and with whom they could discuss different alternatives:

*There are caseworkers who help me find jobs [to apply for], being a reference for me, sending lots of jobs that fit me, interviewing me every week. They work really well with me […] and I’m grateful to them.* (Participant 3, female)

In contrast, dissatisfied participants described difficulties in reaching the PES, an excessive reliance on digital communication, a lack of personal interaction and poor continuity:

*My caseworker is always being changed, but I don’t know why. He doesn’t do anything for me.* (Participant 4, female)

Recognition of professional capabilities and achievements was another important aspect of interactions with authorities. Some participants felt that employment officers and/or supervisors did not fully understand their skills and capabilities. For example, one participant, a trained teacher from her home country, felt that her employment officer thought that immigrants did not understand the Swedish education culture, which was frustrating for her. The interviews indicated that, in some cases, feelings of being misunderstood by employment officer/supervisors could lead individuals to lose confidence in the support offered.

### Being part of a workplace

Participants expressed a strong desire for employment that would provide a sense of meaningful belonging to a workplace. For them, a job was not just about earning a living but also about feeling capable, useful and included in a social context. Although most of their work experiences in Sweden did not fully meet these expectations, the ones that did were important to them. The following sub-themes explore aspects of being part of a workplace as discussed by participants and how these relate to their experiences of work in Sweden.

#### Feeling needed and capable

Participants emphasised the feelings of meaningfulness and being needed by others as important aspects of having a job:

*…because in all these [jobs] I helped people, and my plan was to help people. I have a good life and feel very well here in Sweden. I live because I still help people.* (Participant 6, female)

Participants also emphasised the importance of feeling capable in their work, of mastering the tasks given to them and ensuring that the work aligned with their skills and competencies. Appreciation from employers, colleagues and clients served as an affirmation for many participants, confirming their ability to perform well in their roles.

*I liked the job very much, working with youths and that kind of thing. And it [the work I did] was quite appreciated. I received very good notes and testimonials from my school principal, showing that I worked well.* (Participant 1, female).

Many participants described experiencing a sense of purpose and competence in jobs where they worked with other immigrants as the primary focus as they could make use of their own experiences and language skills in these jobs. For example, one child care worker said she preferred working in suburban areas where many of the children came from immigrant backgrounds. Another participant explained that she had dismissed the idea of pursuing her former career as a journalist, as she would not feel confident working in another language. Instead, she now sought jobs supporting immigrant children and youths:

*[Working] with unaccompanied children, with [people] with other migration [background], that is very good. I have a lot of self-confidence; I can manage myself 100% in these things. Because of that, I look for jobs in schools, in migration, and working with unaccompanied children. That's my hope… - laughs.* (Participant 5, female).

#### Being part of a social context

Being part of a social context was an important aspect of having a job stressed by several participants. Some had experiences of employments who gave them opportunities to make friends with native Swedes and to practise Swedish; others expressed expectations that a job would provide such opportunities. The psychosocial work environment and treatment received from managers and colleagues was also mentioned. One participant, who had worked as a cleaner in a family business, highlighted the importance of being treated with respect and feeling included:

*He [the boss] showed a lot of respect; he did not see me as just a cleaner. He invited me to join a staff trip to Portugal, and each [year] he hosted a Christmas dinner, to which I was also invited.* (Participant 17, male)

Although accounts like the one above illustrates the important role of the social aspect of the work environment, many participants’ descriptions of their work experiences in Sweden notably lacked any mention of colleagues and managers. One possible reason is that working in temporary positions or as substitutes on a day-to-day basis offers few opportunities to forge social ties in the workplace, as reflected upon by this participant:

*[When you work as a substitute] it becomes kind of lonely because those who work there already know each other […]. But you are only there once a week. That's not sufficient to establish contact with them.* (Participant 10, female)

### Balancing aspirations and needs amidst contextual constraints

Findings showed that both individual aspirations and contextual constraints influenced labour market trajectories. The following sub-themes explore these constraints and the different strategies undertaken.

#### Adopting strategies to achieve goals within contextual constraints

Participants described how conflicting needs and limited opportunities made career planning difficult. Although most participants directed their job searches towards one or a few professional areas, they prioritised securing a full-time and/or permanent position over working within a specific profession. In some cases, participants reported opting out of certain jobs due to personal or practical reasons, such as health issues. Many also expressed a desire and plans to pursue further education; however, at the same time, they were hesitant due to financial concerns and their age. Some described working and studying in parallel as an ideal way to solve this dilemma. In practice, though, this was difficult considering the nature of the low-paid and temporary jobs they currently held, a challenge reflected upon by this participant:

*Work comes first for me. I have a family, so I need to help my family. If I got a job, maybe a permanent one, then I could study remotely.* (Participant 14, male)

The strategies adopted by participants depended on the resources available to them and their individual choices, ranging from short-term, ad-hoc decisions to long-term efforts aimed at predefined goals. The accounts revealed that striving towards a long-term goal often entailed handling conflicts and dilemmas. For example, one participant had to decline job offers outside her professional area in favour of a work placement in a relevant field. Another participant had to go against the recommendations of the PES in order to take the necessary steps to validate her qualifications as a teacher. Other participants described their career paths as a more gradual progression, where they set short-term goals and worked towards achieving them one at a time. This was the case for one participant, a warehouse worker, who took a one-day course as a forklift truck driver to improve his chances of securing more working hours at the company. He reasoned that this would eventually enable him to obtain a driver's license, which, in turn, would make his goal of securing a full-time position reachable.

### Navigating aspirations and needs in relation to language skills

Language was an area where conflicting aspirations and needs became evident. On the one hand, participants acknowledged the importance of Swedish language skills for improving job opportunities and expressed a will to improve their Swedish skills. On the other hand, financial pressures meant they had to prioritise job opportunities at hand over language learning. In addition, participants expressed uncertainty about how to improve their language skills, as they felt they had limited opportunities to practise Swedish.

*I try to study Swedish. […] I understand everything, but I don’t practice, there aren’t neighbours, there aren’t any. Even when I went there [job training course], they didn’t speak much Swedish.* (Participant 4, female)

This woman hoped that getting a job and becoming part of a workplace would provide such opportunities, an expectation shared by several of the participants. However, language skills were generally not discussed with employers, and participants were unsure to what extent insufficient language skills was a reason for rejections of applications or not extending job contracts. One exception was a participant who failed to pass an internship at a hospital due to insufficient Swedish language skills. She described that she initially felt very sad and upset, but when she subsequently received the support needed to get approved, her perspective changed. In the end, she felt grateful for the opportunity to improve her language proficiency. In her case, the combination of demands and adequate support helped her to achieve her goals.

Interestingly, a third of the participants had utilised their native language skills as a means of securing employment. These individuals had worked as interpreters, native language teachers, childcare workers, teacher's aides or personal care workers, assisting pupils or clients who shared the same language background. Although some of these positions provided limited opportunities to practise Swedish, participants generally viewed them positively, as they offered job opportunities and work experience. Some also described feeling more at ease working in their native language. However, since the continuation of these positions depended on the demand for specific language skills, they also made participants vulnerable in the labour market.

### Making decisions based on limited information

Participants obtained information about job and education opportunities through their social networks and local communities, as well as from employment officers/supervisors and teachers. Several participants had secured job positions through friends and family. However, others pointed out that the jobs available within their networks were often limited to roles for which they were overqualified.

The participants’ accounts revealed how their knowledge of the labour market changed over time and with experience, as well as how they tried to adapt their strategies in accordance with the insights gained along the way. This was the case for two young women who had studied to become childcare workers in Sweden. They had reasoned that this area would offer opportunities for employment, even though they did not speak Swedish fluently. However, their expectations were not met, as they were only able to get positions as day-to-day substitutes. Both were now considering studying to become nursing assistants instead, an area in which they knew friends with similar background had successfully found jobs. These participants had, to some extent, re-evaluated the importance of education for employment, as they now realised that getting a job also required personal connections. One of the women explained:

*Before, I thought that if you had an education, you would get a job, but now I think that you only get [a job, if you] have someone working there to help you get in.* (Participant 11, female)

By studying to become a nursing assistant, this woman hoped to combine formal education with the connections offered through her personal network, to be able to finally enter the labour market in a more sustainable way.

## Discussion

The aim of this study was to explore immigrants’ experiences of sustainably entering and maintaining employment in the Swedish labour market. The main theme identified was that employment instability over time challenged professional identity, well-being, and sense of agency. For participants in this study, working and being unemployed were not distinct situations; rather, they found themselves in precarious work situations characterised by employment instability, low-income and limited opportunities to enhance employability. Moreover, the findings showed that it was the ongoing nature of this situation that had strong negative effects, both practically and emotionally. An increasing number of studies have shown associations between the flexibilization and precarization of the labour market and negative consequences on workers’ health and well-being.^26–28^ The results from the present study showed that participants’ unstable employment situations led to psychological stress, financial insecurity, and difficulties in planning their lives. It was particularly the prolonged situation of employment instability and lack of career prospects that led to negative emotional consequences, including feelings of frustration and concerns about how deteriorating health, along with increasing age, might further reduce their job opportunities. Unstable and underqualified employment have been suggested to offer a pathway to more stable jobs. This was not, however, the case for participants in this study, a finding that is in line with results from previous qualitative and quantitative research.^8,29,30^ Educational attainment from the country of destination is generally emphasized as a factor promoting immigrants labour market integration. Interestingly, this was not reflected in this study where a majority had gone through vocational education in Sweden within areas with high demand for labour such as health care, building and engineering and education without this leading to a stable employment on the regular labour market. While the findings of this study may enhance general understanding on how the interaction between individual and contextual factors shape the labour market integration process, further research is needed to identify the specific contribution of different factors behind the low pay off from education in this group.

The enduring employment instability experienced by participants in this study calls for a rethinking of the labour market integration process and the support needed from both a systemic and a dynamic perspective, as suggested by the sustainable career conceptual model.^23^ The implications of this will be discussed below in relation to the support offered during the labour market integration process and the influence of enduring employment instability on individuals’ agency and capacity to develop and adapt.

### Supporting labour market participation requires a holistic and flexible approach

Earlier studies showed that immigrants’ labour market integration is not a linear process; rather, it goes back and forth and is heavily dependent on contextual factors and life events.^7,15^ At the same time, labour activation policies often assume a linear transition, which may hinder immigrants from fully benefitting from them.^31^ The results of this study showed that while participants expressed aspirations to create a sustainable working life for themselves, considering aspects such as employment stability and health, however in practice their ability to do so was considerably challenged by contextual constraints. One example of this was that language studies and introduction programmes were often interrupted due to financial needs, family responsibilities or health issues despite participant emphasising the importance of educational efforts and language skills for improving their employment situation. Another factor that influenced participants’ labour market trajectories in this study was the limited access to information on how the labour market functions. Research on career decisions among vulnerable groups has shown that, rather than being rational, decisions tend to be pragmatic or opportunistic, context-related and often involve accepting one option rather than choosing between many, which must be considered when supporting these groups.^32^ Social networks may, for example, facilitate finding a job for disadvantaged groups, however these connections often lead to low-skilled and low-paid jobs that do not promote long-term sustainability.^14,15^ The results of this study highlight the need to improve the information about education and employment alternatives, so that immigrants can make informed decisions based on their individual circumstances.

The lack of improvement in participants’ labour market situations over time in this study raises the question of why their work experiences did not translate into an improved employment situation. One contributing factor to the adverse labour market outcomes of immigrants compared to non-immigrants may be that immigrants do not have the same opportunities for skill development in the workplace, which was shown in one study on work-related health factors for female immigrants in Sweden.^33^ From the present study it is not possible to conclude whether this was the case, however, the finding that work and unemployment often took place in parallel indicates that career support measurements should not be limited to periods of unemployment but should also be provided when immigrants are employed.

### The importance of social support for sustainable labour market participation

Personal agency, flexibility and motivation are important factors on the individual level affecting the chances to get employment.^12,34^ The findings of this study showed that, in addition to the practical implications of participants’ employment situations, the emotional consequences constituted a major challenge for the individual's well-being and sense of agency. The role of identity formation and the influence identity threats may have on individuals’ motivation and career transitions has been studied in previous qualitative research on immigrants’ labour market integration.^14,35^ These studies showed that maintaining a positive self-image was important for an individual's motivation and ability to reorient themselves, and that supportive personal relationships were crucial in this process. Their conclusions regarding the role of social support in identity formation shed light on the findings of the present study, which showed that social support helped participants cope with the negative consequences of unemployment, not least with feelings of frustration and loss of professional identity. This applied not only to support from personal networks but also from teachers, employment officers and employers, illustrated by the importance that participants placed on the relationship with employment officers when evaluating interventions. However, different from what was suggested in previous research,^14,35,36^ the findings of the present study do not seem to support that high career expectations among highly educated immigrants acts as a barrier to labour market participation as most participants were prepared to recalibrate their expectations considering the opportunities available. Rather than high expectations, it was the negative feelings of hopelessness and frustration that, if not dealt with, affected motivation negatively, as well as the ability to act constructively.

A striking result was the participants’ strong desire for meaningful belonging to a workplace. In their vision, employment was not only about earning a living but also about a sense of capability, usefulness and inclusion in a social context. The positive aspects of work expressed by participants largely reflect Jahoda's latent functions of employment (time structure, social context, collective purpose, status and activity) which have been found to play important roles in the relationship between work and health in general populations.^19,37^ The strong desire to belong to a workplace, expressed by participants in this study, indicates that this also holds true for this group. It may even be that for individuals immigrating as adults, the workplace may constitute one of the most important arenas for identity formation and social integration, as pointed out by some researchers.^7,35^

### Learning the language – a multifaceted challenge

The issue of learning the host country language may serve as an illustration of the complexities and dilemmas involved in the labour market integration process. Good skills in the host country language are generally stressed as a crucial factor for successful labour market participation, although too high demands on language skills may also serve as a factor of exclusion.^12,14,38–41^ The results from the present study showed that learning Swedish is a multifaceted challenge. The participants of this study were aware of the importance of language skills for employability and emphasised their willingness to improve their Swedish language skills, however, their accounts also revealed that this ambition may sometimes conflict with other needs and priorities. Most participants had taken courses in Swedish for Immigrants (SFI) which are part of the introduction program offered to newly arrived refugees and family reunification immigrants in Sweden, however, several participants expressed a need for further language training, and some also revealed a lack of confidence in their language skills, indicating that the undertaken courses had not fully met the needs of participants. An increased focus on language training at the workplace has been proposed to improve language training outcomes, although this method also presents challenges.^12,42,43^ A third of the participants in this study had been employed in positions where their native language skills had been requested. In most cases, these positions also required other professional competencies, nevertheless, once the specific language skill was no longer needed, the individual returned to unemployment. Thus, these jobs seemed to offer limited opportunities for individuals to improve their Swedish language skills, although improved this would have enabled the employer to benefit from their competencies in a broader range of positions. The role of language and “multicultural” competences in getting employment was also noted in other studies on immigrants’ labour market establishment.^30,34^ Further studies on this strategy for labour market entry would be important to better understand its’ potential and limitations.

### Methodological considerations

Some considerations are needed regarding the study population and how the results relate to the labour market situation of immigrants in general. First, immigrants form a heterogeneous group, and there is always a risk that applying broad labels may create false generalisations and stigmatisation.^44^ However, the purpose of this study was not to present a representative picture of immigrants as a group, but to increase the understanding of the difficulties faced by immigrants in achieving sustainable labour market participation, as well as the types of support that may be needed. Second, the inclusion criteria were broad, and it is possible that a more narrowly defined population might have enabled a deeper understanding of specific issues. Nevertheless, despite this broad definition, the study identified shared experiences among participants regardless of different education levels and time of immigration. Third, participants were recruited through immigrant organisations, projects and support programmes; thus, it cannot be ruled out that this procedure may have influenced their experiences and, consequently, the results. No major differences were identified between male and female participants regarding the main study questions. However, as there were only a few men in the study and gender differences were not a primary focus of the interviews, it is not possible to draw any firm conclusions on eventual gender differences in relation to the research question.

The impact of power relations on the information collected is another important aspect that needs to be considered when conducting research with vulnerable groups. In the present study, the interviewers’ position as Swedish-born women working on a research project may have influenced the information obtained. To minimise this influence, it was emphasised during the interviews that there were no right and wrong answers and that all experiences were valid and valuable. Another aspect that may have influenced data collection was the choice of Swedish as the interview language, as this could have limited some participants’ ability to fully elaborate their thoughts and feelings. On the other hand, the use of interpreters also has its drawbacks, as it may interfere with the direct interaction between the researcher and the participant.

### Implications for practice and research

The findings of this study have several implications: First, as entering and remaining in the labour market is not a linear process, support needs to be flexible and extended over a long period, adapting to different phases in life. Moreover, support efforts should not be limited to times of unemployment but should also be provided during employment. Second, as supportive social relationships, both in the workplace and outside, are crucial during the process of entering and remaining in the labour market, there is a need to invest more time and effort in the encounters in order to have success with support interventions. Finally, the workplace constitutes a central arena for promoting sustainable labour market participation, and further research is needed to explore how its role can be strengthened. This includes examining how immigrants’ professional development and language skills can be promoted within the framework of their employment, regardless of whether those positions are subsidised or standard, short-term or long-term. Such research should be interdisciplinary, combining knowledge regarding workplace interventions from different disciplines, such as occupational health, adult education and social work.

## Conclusion

The main theme identified was that immigrants’ employment instability over time challenged their professional identity, well-being and sense of agency. The findings showed that, in the process of entering and remaining in the labour market, immigrants had to navigate between their aspirations, needs and contextual constraints while also struggling with feelings of frustration. Social support, both in the workplace and outside, was crucial in coping with this situation.

## Supplemental Material

sj-docx-1-wor-10.1177_10519815261431794 - Supplemental material for First-generation immigrants’ experiences of entering and remaining in the Swedish labour market in a sustainable way: A qualitative study Supplemental material, sj-docx-1-wor-10.1177_10519815261431794 for First-generation immigrants’ experiences of entering and remaining in the Swedish labour market in a sustainable way: A qualitative study by Maria Brendler-Lindqvist, Magnus Svartengren, Martin Tondel and Therese Hellman in WORK
