# Supplementary material for: First-generation immigrants’ experiences of entering and remaining in the Swedish labour market in a sustainable way: A qualitative study
Source: Work. 2026 Mar 18;84(4):1167–80. doi: 10.1177/10519815261431794 (PMC13400809; doi:10.1177/10519815261431794)
Supplement: sj-docx-1-wor-10.1177_10519815261431794 - Supplemental material for First-generation immigrants’ experiences of entering and remaining in the Swedish labour market in a sustainable way: A qualitative study [file sj-docx-1-wor-10.1177_10519815261431794.docx]

**Supplementary_material_appendix_1. Semi-structured interview guide**

**1. Personal background**

-Could you tell me a bit about yourself? Who are you?

-Where are you born and raised?

-When did you come to Sweden? Why did you move here?

-What does your family look like?

-Could you describe your education and work experience before coming to Sweden?

-Do you have any education from Sweden? What work have you done in Sweden?

-What were your thoughts about your future work life in Sweden before you arrived?

*Demographic information to be covered in this section: Age, sex, country of birth, year of immigration, marital status, children, formal education, vocational experience, current labour market situation/duration of unemployment, last employment and current means of subsistence.*

**2. Experiences of employment in Sweden**

-Would you like to tell me about the work you have done in Sweden?

-Which job did you enjoy the most? Why?

*Areas to explore - possible follow-up questions:*

-Could you describe the most positive aspects of your work? Were there things you did not like?

-How was the relationship with your colleagues? How was your relationship with your boss?

-How did you feel the work matched your education and experience?

-Could you explain why you decided to work there? Did you consider other alternatives?

**3. Experiences of termination of employment in Sweden**

-Why did you stop working there? How did that feel?

-When you lost that job, did you apply for similar positions? Why/why not?

-How did you feel when you lost that job and were unable to find another one in the same area? What were you thinking at that time?

**4. Current situation**

-What does your current work situation look like? How does it feel? (What are the main disadvantages? Are there any advantages?)

-How do you feel about not having any colleagues?

-How do you feel about not having a defined place to go to each day?

*Example of follow-up question: You mentioned that it was important for you to help others in you work, how do you feel now when you don’t have any employment?*

**5. Future**

-What do you think about your chances of finding working again? What are the barriers? What opportunities do you see?

-How are you going about finding employment?

-From your perspective, what is the main reason you don’t have a job today? Are there other reasons?

*Areas to explore - possible follow-up questions:*

*-Has your education or vocational background influenced your ability to get and maintain employment? In what way?*

*-Has your health affected your ability to get and maintain employment? How?*

*-Have your family and friends influenced your ability to get and maintain employment? How?*

-Has being foreign-born affected your ability to get and maintain employment? How?

-What kind of support do you feel you need?

*Possible follow-up questions: From society (which stakeholders)? From family and friends?*

-If you think back on your working life, is there anything you would have done differently?

-How do you think your life will look like in ten years?

**Overall reflections:**

-In addition to the issues we have touched upon already, do you have other ideas on how a long-term sustainable working life could be facilitated for foreign-born individuals?

-Would you like to add anything regarding your working life experience in Sweden?

-May we contact you for follow-up questions if necessary?
